# Genetic genealogy of Y-chromosome in the Zhetiru tribe of the Kazakh population from Kazakhstan

**DOI:** 10.3389/fgene.2025.1516130

**Published:** 2025-03-24

**Authors:** Aigul Zhunussova, Saltanat Tayshanova, Alizhan Bukayev, Ayagoz Bukayeva, Baglan Aidarov, Radik Temirgaliev, Zhaxylyk Sabitov, Maxat Zhabagin

**Affiliations:** ^1^ Astana International University, Astana, Kazakhstan; ^2^ National Center for Biotechnology, Astana, Kazakhstan; ^3^ Research Institute for Jochi Ulus Studies, Astana, Kazakhstan; ^4^ Kazak Historical Society, Astana, Kazakhstan; ^5^ DNK Shejire LLP, Astana, Kazakhstan

**Keywords:** Y-chromosome, STR (short tandem repeat), haplotype diversity, genetic genealogy, Zhetiru tribe, Kazakh population, population genetics

## Abstract

**Introduction:**

The Y chromosome, transmitted exclusively through the paternal line, is a well-established tool for verifying genealogical data. The Kazakh tribe Zhetiru in Kazakhstan, comprising seven clans, has conflicting historical and genealogical narratives regarding its origin—either as a union of seven independent clans or as descendants of a single common ancestor. A detailed genetic investigation has not yet addressed this question.

**Methods:**

350 male volunteers from the Zhetiru tribe were analyzed using 23 Y-STR loci and 17 Y-SNPs. We calculated genetic distances using Arlequin and STRAF, and explored genetic structure with median-joining networks using a comparative dataset of over 3,000 Kazakh individuals.

**Results:**

At the tribal level, haplotype diversity (0.997) and haplogroup diversity (0.91) are high. However, at the clan level, haplotypic diversity decreases, revealing clear founder effects in the main haplogroups of Kerderi (R1a1a), Kereit (N1a2), Tama (C2a1a3), and Teleu (J2a2). The genetic structures of Zhagalbaily, Ramadan, and Tabyn indicate additional sub-clan founders. The ages of key clusters suggest stable genetic lineages for over 1,000 years. Zhetiru clans do not form a distinct genetic cluster among Kazakh tribes but demonstrate genetic affinities with others.

**Conclusion:**

This study demonstrates the effective application of genetic genealogy approaches in verifying historical and genealogical records concerning the Zhetiru tribe and determining its origin from distinct, genetically independent clans.

## 1 Introduction

The Y chromosome has proven to be a unique source of genealogical information that can fill gaps in family history that cannot be reconstructed using traditional archival documents (Calafell and Larmuseau, 2017). The fact that the Y chromosome can only be passed down through males makes it possible to find very accurate biological relationships. This is especially important when looking at the common ancestors of Central Asian tribal groups (Chaix et al., 2004).

The Kazakhs represent one of the most complex and detailed tribal systems of the Eurasian steppe. Their traditional genealogy, termed shezhire, comprises intricate records transmitted through generations along the patrilineal line, connecting clan members to a shared progenitor. Scholars have primarily viewed the Kazakh shezhire as a social construct, largely ignoring the genetic foundations of these connections. Recent genetic research (Zhabagin et al., 2021; Khussainova et al., 2021; Zhabagin et al., 2024) shows that traditional Kazakh tribal groups are mostly the same as patterns found by Y-chromosome analysis. The verification of genealogical data for Kazakh tribal groups via Y-chromosome analysis is becoming crucial in the examination of Kazakh ethnogenesis.

Although Y-chromosome analysis is highly informative for studying patrilineal relationships, this approach has certain limitations. Specifically, it does not provide insights into matrilineal connections or genome-wide genetic diversity, which can be explored through autosomal markers. However, the choice of Y-chromosome as the primary tool in this study is justified, as it allows for the most precise assessment of patrilineal relationships, which is particularly crucial for verifying the Kazakh shezhire (genealogy).

**FIGURE 1:**
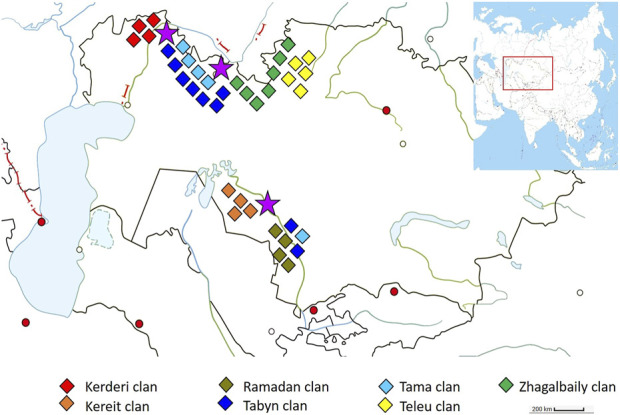
The settlement of the Zhetiru tribe during the early 20th century. The color rhombuses serve as indicators of the settlement regions for the seven clans. The purple stars indicate the averaged geographical locations where the samples were collected.

One of these tribes is the Zhetiru, which is part of the Kazakh people and numbered about 374 thousand at the beginning of the 20th century (Temirgaliyev, 2010). Since then, national population censuses in Kazakhstan have not recorded data on tribal and clan affiliation among Kazakhs. The name of the tribe comes from the Kazakh words “zhety” (seven) and “ru” (clan), which reflects its genealogical structure, consisting of seven clans: Zhagalbaily, Kerderi, Kereit, Ramadan, Tabyn, Tama, and Teleu. The main settlements of the Zhetiru tribe are located on the territory of the modern Aktobe and Kyzylorda regions of Kazakhstan (Figure 1).

In 1748, Tevkelev wrote the first record of the Zhetiru tribe’s creation (Erofeeva IV, 2005). According to his records, in the second half of the 17th century, seven independent clans formed a political tribal union called “Zhetiru.” However, based on materials collected in the 1820s among Kazakh elders, Blumberg proposed a different version of the Zhetiru tribe’s origin, claiming that it descended from a single ancestor, Karakatysh, who had seven sons, each of whom founded one of the seven clans (Blaramberg, 1848). Karakatysh is regarded Alshyn’s third son and the ancestor of the Kazakh tribes Alimuly and Bayuly. Sultanov’s lists of 92 nomadic tribes of the Ulus of Jochi, including Majmu’ at-Tavarikh (16th century), lend credibility to this story. He points out that the Tabyn, Tama, Ramadan, Teleu, and Kerderi clans (save for Zhagalbaily) are mentioned together in these lists, implying the presence of stable relationships, including genetic ones, between these clans as early as the 16th century (Sultanov 1982).

Only two studies (Zhabagin et al., 2021; Ashirbekov et al., 2022a) have performed genetic analyses of the Zhetiru tribe, mainly focusing on the population structure of Kazakhs in western Kazakhstan. Nevertheless, these studies did not thoroughly examine the origins of the Zhetiru tribe, resulting in a deficiency in comprehending their genetic lineage. This study aims to ascertain two hypotheses on the origin of the Zhetiru tribe (whether from a single common ancestor or many origins) and to establish the genetic positioning of seven clans within the tribe relative to other Kazakh tribes. This study presents the inaugural characterization of Y-chromosome polymorphism in the seven clans of the Zhetiru tribe through the investigation of 23 Y-STR and Y-SNP markers.

## 2 Materials and methods

### 2.1 Sample and data collection

The study was conducted in accordance with the Declaration of Helsinki: Ethical Principles for Medical Research Involving Human Subjects. Adopted in 1964 and was approved by the Institutional Review Board (or Ethics Committee) of the National Center for Biotechnology (protocol code №5, dated 16 October 2020).

The study recruited unrelated healthy male volunteers of Kazakh descent from Kazakhstan. Each volunteer provided informed consent by signing a consent form and completed an ethnographic questionnaire, which included information about their tribal and clan affiliation. We collected saliva samples from 350 males of the Zhetiru tribe using the Oragene DNA Self-Collection Kit (OG-500, DNA Genotek, Canada). As a result, seven clans of the Zhetiru tribe were collected for the study: Kerderi clan (N = 40), Kereit clan (N = 32), Ramadan clan (N = 39), Tabyn clan (N = 85), Tama clan (N = 36), Teleu clan (N = 53), and Zhagalbaily clan (N = 65). Population genetic data are elaborated in Supplementary Table S1.

### 2.2 DNA isolation, amplification and STR genotyping

DNA extraction from saliva samples was performed utilizing the prepIT-L2P kit (DNA Genotek, Canada). Following isolation, DNA concentrations were measured using a Qubit 2.0 Fluorometer (Thermo Fisher Scientific, United States) with the Qubit dsDNA BR Assay Kit (Thermo Fisher Scientific, United States). The integrity and purity of DNA were assessed using NanoDrop One Spectrophotometry (Thermo Fisher Scientific, United States). PCR amplification was performed utilizing the PowerPlex Y23 System (Promega, United States) on a SimpliAmp Thermal Cycler (Thermo Fisher Scientific, United States). The electrophoretic separation of PCR products was performed utilizing the WEN Internal Lane Standard 500 (Promega, United States) in Hi-Di Formamide (Thermo Fisher Scientific, United States) with an 8-capillary Applied Biosystems 3,500 genetic analyzer, which was equipped with POP-4 polymer and Cathode and Anode buffers (Thermo Fisher Scientific, United States). Control DNA 007 (Thermo Fisher Scientific, United States) was utilized as the positive control, while ddH2O was used as the negative control for each batch of Y-STR fragment analysis. The PowerPlex Y23 System (Promega, United States) comprises 17 typical Y-STR markers (DYS19, DYS385 a/b, DYS389I/II, DYS390, DYS391, DYS392, DYS393, DYS437, DYS438, DYS439, DYS448, DYS456, DYS458, DYS635, Y-GATA-H4) and 6 loci characterized by elevated mutation rates (DYS481, DYS533, DYS549, DYS570, DYS576, DYS643). Samples exhibiting non-standard patterns, off-ladder alleles, or microvariant alleles were re-evaluated. Our laboratories have successfully completed the YHRD Quality Control Test (YC000343) and provided haplotype data accordingly. In accordance with the population genetic data rules (Gusmão et al., 2017), the haplotypes were submitted to the Y-Chromosome Haplotype Reference Database (Willuweit and Roewer, 2015) (YHRD, http://www.yhrd.org) with accession number YA006029. Genotyping was carried out using 17 Y-SNPs candidate for core haplogroups (F1756, M48, F1918, F1067, M174, M123, M285, M438, CTS7683, PF5050, M178, CTS6380, M122, M346, M198, M478, M269) on a QuantStudio5 instrument (ThermoFisher Scientific, United States) using TaqMan assays (ThermoFisher Scientific, United States).

### 2.3 Population genetic data

We compiled a dataset for comparative analysis, which included 3,036 Kazakh samples from 20 major tribal groups. Alban (N = 68), Alimuly (N = 283), Argyn (N = 346), Baiuly (N = 572), Dulat (N = 261), Kanly (N = 70), Kerey (N = 154), Konyrat (N = 269), Kozha (N = 88), Kypshak (N = 37), Naiman (N = 162), Oshakty (N = 57), Shanyshkyly (N = 36), Shaprashty (N = 38), Suan (N = 49), Sunak (N = 35), Syrgeli (N = 48), Yssty (N = 72), Zhalayr (N = 210), Zhetiru (N = 181), previously studied for at least 17 Y-STR (Abilev et al., 2012; Balanovsky et al., 2015; Zhabagin et al., 2017; Zhabagin et al., 2019; Zhabagin et al., 2020; Zhabagin et al., 2021; Wen et al., 2020; Khussainova et al., 2021; Ashirbekov et al., 2022b; Ashirbekov et al., 2023).

### 2.4 Data analysis

We analysed STR allele calls from electropherograms using the GeneMapper IDx v.1.6 software (Thermo Fisher Scientific, United States). Haplotype frequencies were determined through the Arlequin program version 3.5.2.2 (Excoffier and Lischer, 2010). We directly calculated the number of distinct haplotypes, the frequency of unique haplotypes, discrimination capacity, haplotype match probability, and haplotype diversity using Microsoft Office Excel. The formula used to find haplotype diversity (HD) is HD = n*(1 − ∑pi ^ 2)/(n − 1), where n is the sample size and pi is the frequency of the *i*th haplotype (Nei and Tajima, 1981). Haplotype match probability (HMP) was calculated as the sum of the squared observed haplotype frequencies. Discrimination capacity (DC) was defined as the ratio of the number of distinct haplotypes to the total number of haplotypes. We used the STRAF 2.1.5 software (Gouy and Zieger, 2017) to calculate forensic parameters such as Random Match Probability (RM), Power of Discrimination (PD), Gene Diversity (GD), Polymorphism Information Content (PIC), Power of Exclusion (PE), Typical Paternity Index (TPI), and the frequency for each locus. This software also facilitated the illustration of Nei’s genetic distances (Nei, 1987) through neighbor-joining (N-J) tree and multidimensional scaling (MDS). Pairwise genetic distances (R_ST_) were computed using the “AMOVA and MDS” online tool on the Y-Chromosome Haplotype Reference Database website (http://www.yhrd.org). Genetic differentiation within and among groups of populations (AMOVA) was performed in Arlequin program version 3.5.2.2 (Excoffier and Lischer, 2010). Median-joining networks were constructed using NETWORK v10.2.0.0 and NETWORK Publisher v2.1.2.5 (Bandelt et al., 1999). Intermediate alleles with repeat numbers were rounded to the nearest integer, and the DYS385a/b loci were excluded from network construction due to the inability to associate specific alleles with their respective copies. We assessed haplotype affiliation to haplogroups using the Nevgen Y-DNA haplogroup predictor (https://www.nevgen.org/) to facilitate subsequent genotyping of Y-SNPs for the core haplogroups. TMRCA determined using the rho-statistic (Forster et al., 1996; Saillard et al., 2000; Macaulay et al., 2019). The mutation rate of 2.1 × 10^−3^ mutations per STR per generation was used (Ge et al., 2009). The generation time was set to 30 years (Fenner, 2005).

## 3 Results and discussion

### 3.1 Haplotype/allele frequencies and forensic parameters

Supplementary Table S1 presents the distribution of 23 Y-STR haplotypes among 350 Kazakh individuals from the Zhetiru tribe. Table 1 details the identification of 260 distinct haplotypes from these 350 samples. Out of these, 215 haplotypes (61%) were unique, and at least two individuals shared the remaining 45 haplotypes. The most frequent haplotype was observed in 9 individuals, while 23 haplotypes were shared by 2 individuals each. The calculated values for haplotype diversity (HD), discrimination capacity (DC), and haplotype match probability (HMP) were 0.997, 74%, and 0.006, respectively (Table 1). Comparing these data with those from Kazakh subpopulations in various regions (Ashirbekov et al., 2024), it is evident that the tribal level demonstrates equal genetic diversity. The reduction in diversity becomes more pronounced at the clan level (Table 1). The Zhagalbaily and Tabyn clans exhibit the highest haplotypic diversity, albeit not surpassing the overall diversity of the Zhetiru tribe. These results show that the PowerPlex Y23 System loci panel is not very good at telling the difference between closely related paternal lineages in the Zhetiru tribe and its clans.

**TABLE 1:** Comparison of genetic polymorphism of 23 Y-STR haplotypes within the Zhetiru tribe.

Population	Number of samples	Number of distinct haplotypes	Frequency of unique haplotypes	Discrimination capacity	Haplotype match probability	Nei’s haplotype diversity
Zhetiru tibe	350	260	61%	74%	0.006	0.997
Kerderi clan	40	26	50%	65%	0.064	0.960
Kereit clan	32	19	47%	59%	0.117	0.911
Ramadan clan	39	28	59%	72%	0.053	0.972
Tabyn clan	85	69	71%	81%	0.019	0.993
Tama clan	36	31	75%	86%	0.037	0.990
Teleu clan	53	33	43%	62%	0.045	0.973
Zhagalbaily clan	65	57	80%	88%	0.021	0.995

**FIGURE 2:**
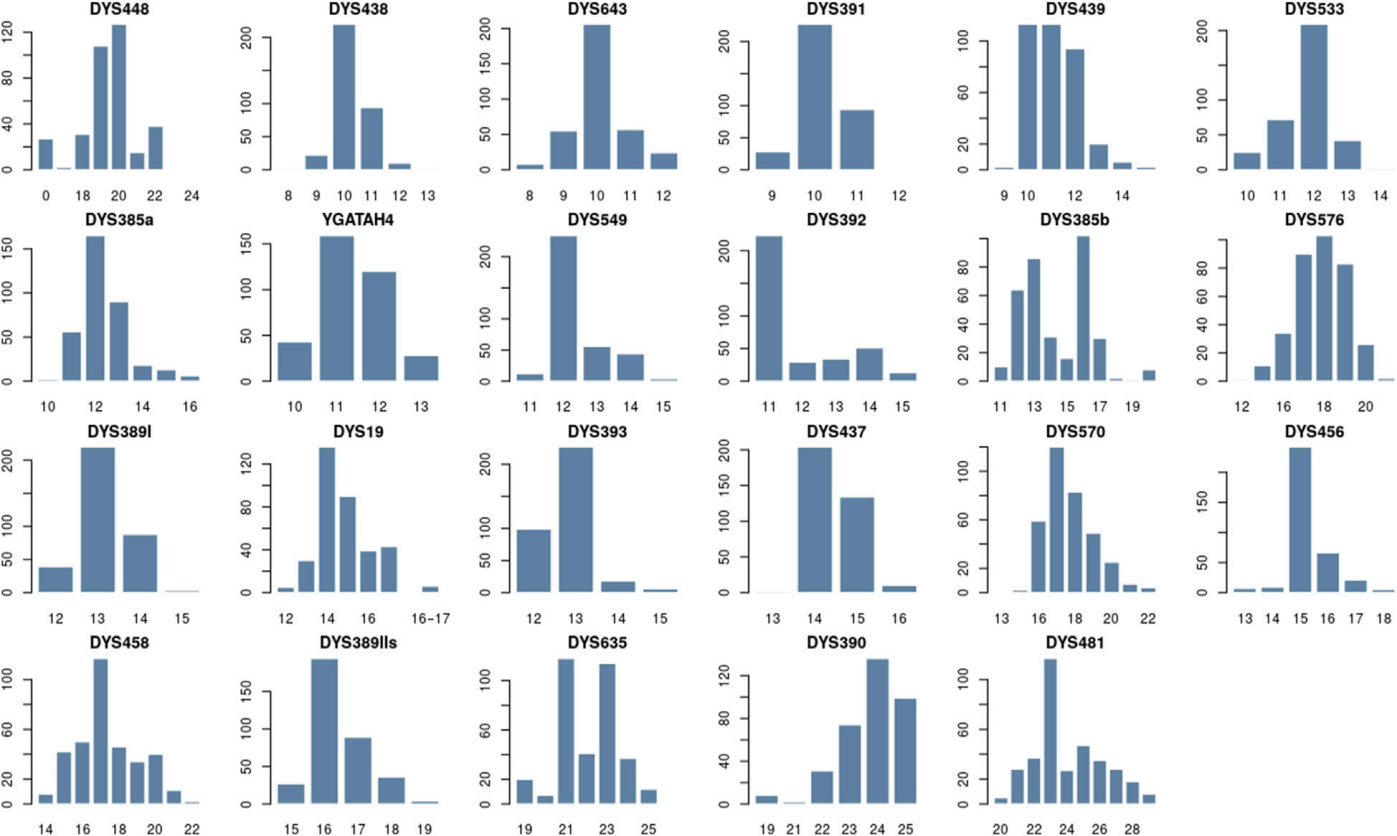
Distribution of allelic frequencies per locus on 23 Y-STR loci in Zhetiru tribe form Kazakh population using STRAF software.

Figure 2 presents the distribution of allele frequencies and forensic parameter values for 23 Y-STRs of the Zhetiru tribe in Supplementary Tables S2, S3. We identified 131 alleles across the single-copy loci, with frequencies ranging from 0.01 to 0.69. The loci DYS389I, DYS391, DYS393, DYS437, and YGATAH4 exhibited the lowest variability, each presenting four alleles. Among these, DYS393 showed the lowest gene diversity (GD = 0.50), while DYS481 was the most polymorphic, exhibiting 10 alleles and a GD of 0.83. For the multi-copy locus DYS385, 30 allele combinations were detected, with a GD of 0.89. Supplementary Table S4 shows that there are some unusual alleles. For example, seven samples have two sets of alleles at locus DYS19, and 27 people have a null allele at locus DYS448.

### 3.2 Haplogroup frequencies and median-joining networks

Table 2 presents the Y-chromosome haplogroup distribution for the Zhetiru tribe. The tribe exhibits a high level of haplogroup diversity (HD = 0.91). It indicates a complex genetic landscape, reflective of multiple ancestral paternal lineages contributing to the gene pool of the tribe. Nine haplogroups, each with a frequency greater than 5%, concentrate the majority of the Y-chromosome variation (86%). These haplogroups include C2a1a1b1 (7%), C2a1a2 (10%), C2a1a3 (10%), J2a1 (6%), J2a2 (13%), N1a2 (10%), O2 (8%), R1a1a (14%), and R1b1a1a1 (8%). Within the seven clans of the Zhetiru tribe, certain haplogroups accumulate at even higher frequencies, and some become clan-specific with frequencies exceeding 50%. Specifically, the Teleu clan predominantly exhibits haplogroup J2a2 (83%), the Tama clan is characterized by C2a1a3 (64%), the Kereit clan shows a high frequency of N1a2 (59%), and the Kerderi clan primarily has R1a1a (55%). The Ramazan (HD = 0.81), Tabyn (HD = 0.82), and Zhabagaly (HD = 0.79) clans exhibit high haplogroup diversity without a major haplogroup (frequency less than 50%).

**TABLE 2:** Frequencies of Y-chromosomal haplogroups within the Zhetiru tribe.

Core Haplogroups	Zhetiru tirbe	clans						
		*Kerderi*	*Kereit*	*Ramadan*	*Tabyn*	*Tama*	*Teleu*	*Zhagalbaily*
	N = 350	N = 40	N = 32	N = 39	N = 85	N = 36	N = 53	N = 65
C2a1a1b1-F1756	0.071	0.000	0.031	0.026	0.259	0.000	0.000	0.015
C2a1a2-M48	0.103	0.075	0.125	0.026	0.259	0.111	0.019	0.015
C2a1a3-F1918	0.100	0.025	0.063	0.026	0.071	0.639	0.000	0.031
C2b-F1067	0.009	0.025	0.000	0.000	0.000	0.000	0.000	0.031
D1-M174	0.003	0.000	0.000	0.000	0.000	0.000	0.000	0.015
E1b1b1b2a1-M123	0.011	0.000	0.000	0.000	0.000	0.111	0.000	0.000
G1-M285	0.014	0.000	0.000	0.026	0.012	0.028	0.000	0.031
I2a-M438	0.017	0.000	0.000	0.000	0.000	0.000	0.000	0.092
J2a1-CTS7683	0.063	0.300	0.188	0.000	0.024	0.028	0.000	0.015
J2a2-PF5050	0.131	0.000	0.000	0.000	0.000	0.000	0.830	0.031
N1a1a-M178	0.017	0.000	0.000	0.000	0.000	0.000	0.075	0.031
N1a2-CTS6380	0.100	0.000	0.594	0.000	0.176	0.000	0.000	0.015
O2-M122	0.077	0.000	0.000	0.205	0.000	0.028	0.000	0.277
Q1b-M346	0.026	0.000	0.000	0.231	0.000	0.000	0.000	0.000
R1a1a-M198	0.143	0.550	0.000	0.308	0.141	0.028	0.019	0.031
R1b1a1a1-M478	0.080	0.025	0.000	0.026	0.012	0.000	0.038	0.354
R1b1a1b-M269	0.034	0.000	0.000	0.128	0.047	0.028	0.019	0.015
Haplogroup diversity	0.91	0.62	0.61	0.81	0.82	0.58	0.31	0.79

Central and East Asia predominantly hosts the Y-chromosome haplogroups C2a1a1b1, C2a1a2, and C2a1a3. C2a1a1b1 is particularly associated with Mongolic and Turkic populations, reflecting historical expansions, such as the Mongol Empire (Wei et al., 2017; Zhang et al., 2018; Wei et al., 2018; Sun et al., 2021). Siberian and Mongolian populations, as well as Turkic speakers, prominently display C2a1a2 (Liu et al., 2021; Zhabagin et al., 2021). These haplogroups exemplify the complex population movements across the Eurasian Steppe, especially during the Bronze and Iron Ages. At the regional level, haplogroup C2 exhibits distinct geographic patterns and strong associations with specific Kazakh tribal groups, reflecting historical migration events and demographic processes. In Western Kazakhstan, the Alshyn tribe shows a predominance of the C2a1a2 lineage (87%), which is linked to expansions into the Caspian steppe. In contrast, the Uysun tribe in southern Kazakhstan exhibits a high frequency of C2a1a3 (40%), suggesting genetic continuity with early Niru’un Mongols. The Konyrat tribe, historically connected to Mongolic polities, demonstrates a striking prevalence of the C2b1a1a1a subclade (86%). These distribution patterns reinforce the role of haplogroup C2 as a genetic marker of steppe nomadic expansions across Central Asia. The Neolithic saw the expansion of J2a1 and J2a2, which originated in the Fertile Crescent (Sahakyan et al., 2021), while N1a2 is associated with Uralic-speaking populations of Siberian origin (Ilumäe et al., 2016). O2 is widely distributed in East Asia, particularly in China, due to the Neolithic agricultural transition, which originated in the Yangtze and Yellow River basins (Yan et al., 2014; Wang et al., 2024). Indo-European migrations link R1a1a and R1b1a1a1; R1a1a is associated with the expansion of Indo-Iranian language (Underhill et al., 2015), while R1b1a1a1 is associated with both European and Central Asian Bronze Age cultures (Myres et al., 2011).

**FIGURE 3:**
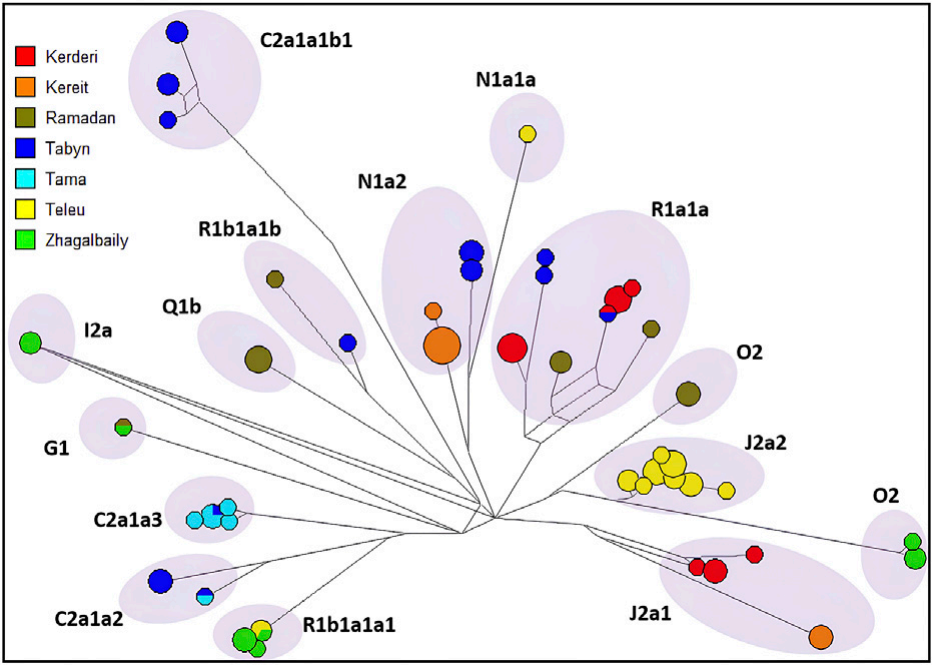
Median-joining network for the haplotypes 350 Kazakh from Zhetiru tribe, constructed from data on 21 Y-STRs. The colours representing the seven clans. Circles represent haplotypes (Frequency > 1 criterion active), with the area proportional to sample size, and lines between them proportional to the number of mutational steps.

The distribution of haplotypic diversity within seven clans of the Zhetiru tribe (Kerderi, Kereit, Ramadan, Tabyn, Tama, Teleu, Zhagalbaily) is visualized using a median-joining network for 21 Y-STRs, as shown in Figure 3. Multiple clusters associated with haplogroups and specific clans were identified. A strong single-founder effect is characteristic of the Tama and Teleu clans, indicating descent from a single paternal ancestor. In contrast, the Tabyn clan is composed of at least five distinct genetic founding lineages. Despite the observed reduction in genetic diversity at the clan level, it is essential to consider potential confounding factors such as recent inter-clan admixture and possible sampling bias. Participant selection was based on self-identification according to the traditional Kazakh genealogical system (shezhire); however, individual cases of recent inter-clan gene flow cannot be entirely ruled out. Future studies incorporating autosomal data will provide a more comprehensive assessment of the extent of recent admixture among clans.

We constructed median-joining networks, as shown in Supplementary Figure S1, to estimate the time to the most recent common ancestors of the founder clans within the Zhetiru tribe and to assess inter-clan relationships for the nine most frequent Y-chromosome haplogroups. These networks visually illustrate haplotype relationships, highlighting patterns of genetic divergence and connections among the clans. For haplogroups such as C2a1a2, N1a2, O2, R1a1a, and R1b1a1a1, at least two distinct clusters were identified. For example, within R1a1a, the Kereit clan shows two genealogical lines, likely reflecting different sub-clan founders. The Zhagalbaily clan displays two distinct genealogical lines within R1b1a1a1 and one within O2. The TMRCA estimates for the major clusters suggest that these clans have maintained stable genetic lineages for at least 1,000 years. This observation is consistent with previous findings indicating an expansion of multiple minor lineages over the past millennium, potentially reflecting the historical and demographic processes that shaped modern Central Asian populations (Zhabagin et al., 2022). Further research at the sub-clan level, leveraging extended Y-chromosome sequencing with tools such as BigY700, is necessary to refine TMRCA estimates and provide deeper insights into the genetic genealogy of the Zhetiru tribe. Such analyses will help contextualize the historical and evolutionary significance of these findings.

### 3.3 Genetic differentiation among Kazakh tribes

To assess genetic differentiation within and among groups of the studied clans and to identify the driving forces shaping Y-chromosome haplogroup variation within the Zhetiru tribe, we performed an Analysis of Molecular Variance (AMOVA) using different criteria for clustering the clans (Supplementary Table S5). The first grouping considered all seven clans to evaluate molecular variation among clans. The second grouping was based on geographic distribution, following historical records of clan settlement patterns. We provisionally categorized the clans into two geographic groups: northern (Kerderi, Teleu, Zhagalbaily, Tabyn, and Tama) and southern (Kereit and Ramadan). Although certain sub-clans of Tabyn and Tama are also found in the south, they predominantly inhabit northern regions and were therefore assigned to the northern group for this analysis.

The results indicate moderate genetic differentiation among clans (FST = 0.29), confirming that the clans are genetically structured and exhibit distinct genetic variation. However, there is no significant genetic differentiation between the two geographic groups (FCT = −0.07), suggesting that genetic variance between the northern and southern groups was either absent or lower than expected under random distribution. The negative FCT value (−0.07) indicates a lack of significant geographic structure among the Zhetiru clans. This result can be attributed to the high mobility of Kazakh tribes, driven by historical migrations. Furthermore, the highest genetic diversity was observed at the within-population level, indicating that most genetic variation is found within individual clans rather than between them. These findings suggest that clan-based genetic structure is more biologically meaningful than geographic grouping, as historical tribal organization appears to have played a more significant role in shaping Y-chromosome variation within the Zhetiru tribe.

Supplementary Table S6 presents the calculated pairwise genetic distance (R_ST_) between Kazakh tribes and Zhetiru clans based on 17 Y-STR markers. The Argyn tribe (mean d = 0.56), the Konyrat tribe (mean d = 0.47), and the Kerei tribe (mean d = 0.47) exhibit the most distant genetic relationships from all Zhetiru clans, except for the distance between the Tama clan and the Kerei tribe (d = 0.09). The Tama clan shows genetic proximity to the clans of the Uysun tribe: Alban (d = 0.01), Dulat (d = 0.03), Oshakty (d = 0.06), Shanshkily (d = 0.05), Shapyrashty (d = 0.08), and Suan (d = 0.01), with the exception of the Syrgeli (d = 0.41) and Ysty (d = 0.26) clans. For both of these clans, the closest Zhetiru clan is Kereit (d = 0.1 and d = 0.2, respectively). The most genetically distant Zhetiru clan from the other Kazakh tribes and clans is Teleu (mean d = 0.49), with the smallest distance observed from the Jalair tribe (d = 0.28). The second most distant clan is Zhagalbaily (mean d = 0.34), with the closest genetic relationships being with Kanly (d = 0.20) and Sunak (d = 0.20). Recognized as Islamic missionaries, the Sunak and Kozha clans display close genetic distances to the Tabyn clan (d = 0.07 and d = 0.05), Ramadan (d = 0.15 and d = 0.15), and Kerderi (d = 0.14). The Jalair tribe also shows proximity to the Tabyn clan (d = 0.07) and Ramadan (d = 0.15). However, the closest genetic relationship for the Ramadan clan is with the Kanly tribe (d = 0.13). The very close genetic distances between the clans suggest a common paternal origin, which requires further confirmation through detailed phylogenetic tree analysis and correlation with historical and genealogical evidence.

**FIGURE 4:**
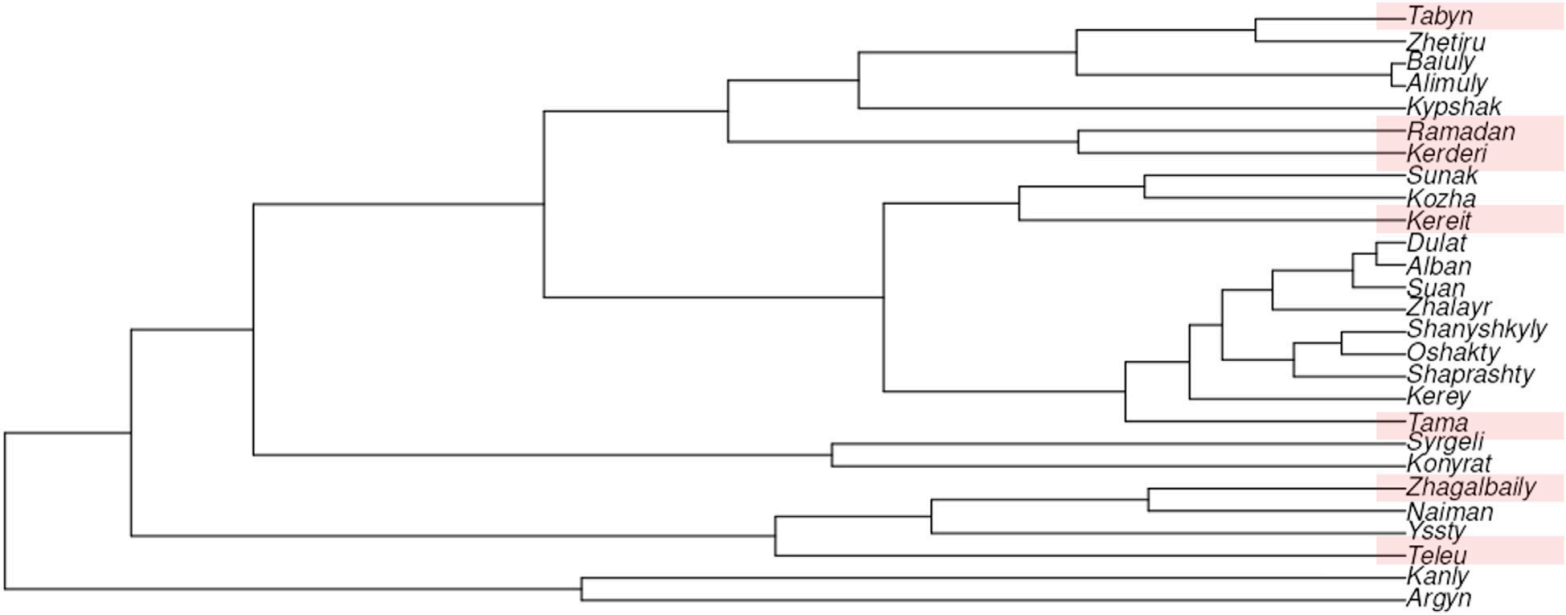
The neighbor-joining tree based on Nei’s genetic distances between Kazakh populations on 17 Y-STRs.

We used Nei’s genetic distances for 17 Y-STR loci to construct a neighbor-joining (N-J) tree (Figure 4). The results clearly demonstrate the genetic differentiation among the clans of the Zhetiru tribe, as well as their genetic affinities with other Kazakh tribes. In addition to the seven Zhetiru clans analyzed in this study, the phylogenetic tree includes a previously published Zhetiru samples, which appears to be predominantly represented by individuals from the Tabyn clan, as they cluster together in a single lineage.

A distinct cluster is also formed by the Kerderi and Ramadan clans, despite their geographic separation, with Kerderi inhabiting the extreme northwestern region and Ramadan being located in the southeastern part of Kazakhstan. The Kereit clan, in contrast, clusters phylogenetically with Kozha and Sunak, both of which are historically associated with the steppe clergy, highlighting a potential historical or social structuring in their genetic relationships.

The Tama clan clusters with a larger group composed of Uisun, Kerey, and Zhalayr, tribes that historically played a central role in the Mongol Empire and its legacy. Similarly, the Zhagalbaily clan exhibits a close phylogenetic relationship with the Naiman, whereas the Teleu and Ysty clans form a distinct and cohesive cluster of their own.

These findings provide valuable insights into the historical genetic structure of Kazakh tribes, emphasizing that the observed clustering patterns are shaped by both genealogical heritage and historical socio-political interactions within the broader context of the Eurasian steppe.

Additionally, we visualized the results using an MDS plot (Supplementary Figure S2), which illustrates the genetic positioning of the Zhetiru clans within the genetic landscape of Kazakh tribes. We can conditionally divide this genetic space into three clusters. The first, includes the Alshin clans of Alimuly and Baiuly, as well as one of the Zhetiru clans, the Kerderi. The second, comprises the majority of the Uysun tribe clans, the Kerey tribe, and three Zhetiru clans—Kereit, Tabyn, and Tama. The Zhetiru clan Zhagalbaily, along with the Konyrat and Naiman tribes and the Yssty clan, forms the third cluster. The Argyn tribe, the Syrgeli clan, and two Zhetiru clans—Ramadan and Teleu—occupy distinct positions within the genetic space. Thus, for the Zhetiru tribe, we observe at least five sources of paternal genetic heritage.

## 4 Conclusion

This study highlights the overall haplogroup frequencies, haplotype structure, and diversity within the Zhetiru tribe. It provides valuable insights into verifying historical and genealogical records related to the tribe and establishing its origins from genetically independent clans. At the same time, the research defines the position of individual clans of the Zhetiru tribe within the genetic landscape of the Kazakh tribal structure.

By analyzing 23 Y-STR loci and 17 Y-SNPs from 350 individuals, we have demonstrated that the seven clans constituting the Zhetiru tribe originate from genetically independent founding lineages rather than a single paternal ancestor. The high haplotype diversity observed at the tribal level, coupled with a significant reduction at the clan level, suggests strong founder effects in multiple clans (Tama and Teleu), while others exhibit more complex multi-lineage structures. Our findings support historical accounts describing the Zhetiru tribe as a union of previously distinct clans. Additionally, the genetic affinities observed between some clans and other Kazakh tribes provide a foundation for further exploration of historical intertribal interactions and population dynamics that have shaped the contemporary tribal landscape. These findings contribute to a deeper understanding of the fine-scale genetic structure of Kazakh tribes.

Putting together traditional genealogical information (shezhire) with results from Y-chromosome population genetic studies helps us learn more about how tribes are organized and how they have changed over time. This interdisciplinary approach not only helps clarify genealogical connections but also identifies key migration patterns and demographic events that have significantly shaped the formation of modern populations. The combination of cultural traditions and genetic data contributes not only to the restoration of historical memory but also holds importance for modern anthropological and historical research, as well as for applied aspects in forensic investigations.

## Data Availability

The datasets presented in this study can be found in online repositories. The names of the repository/repositories and accession number(s) can be found in the article/Supplementary Material.
