# Supplementary figures and images for: Genetic genealogy of Y-chromosome in the Zhetiru tribe of the Kazakh population from Kazakhstan
Source: Front Genet. 2025 Mar 24;16:1516130. doi: 10.3389/fgene.2025.1516130 (PMC11973391; doi:10.3389/fgene.2025.1516130)

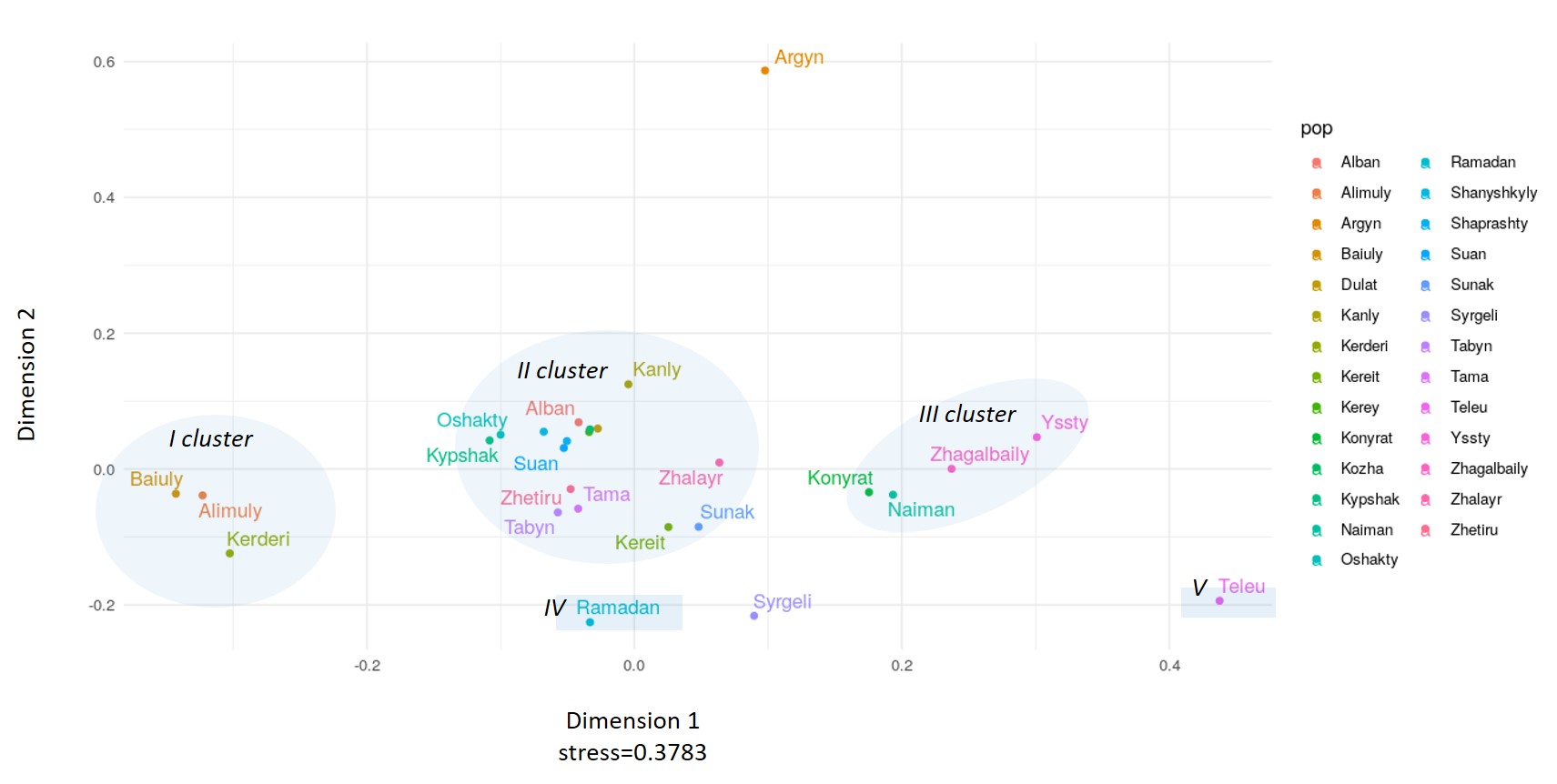

Supplement: Supplementary file 1 [file Image2.JPEG]

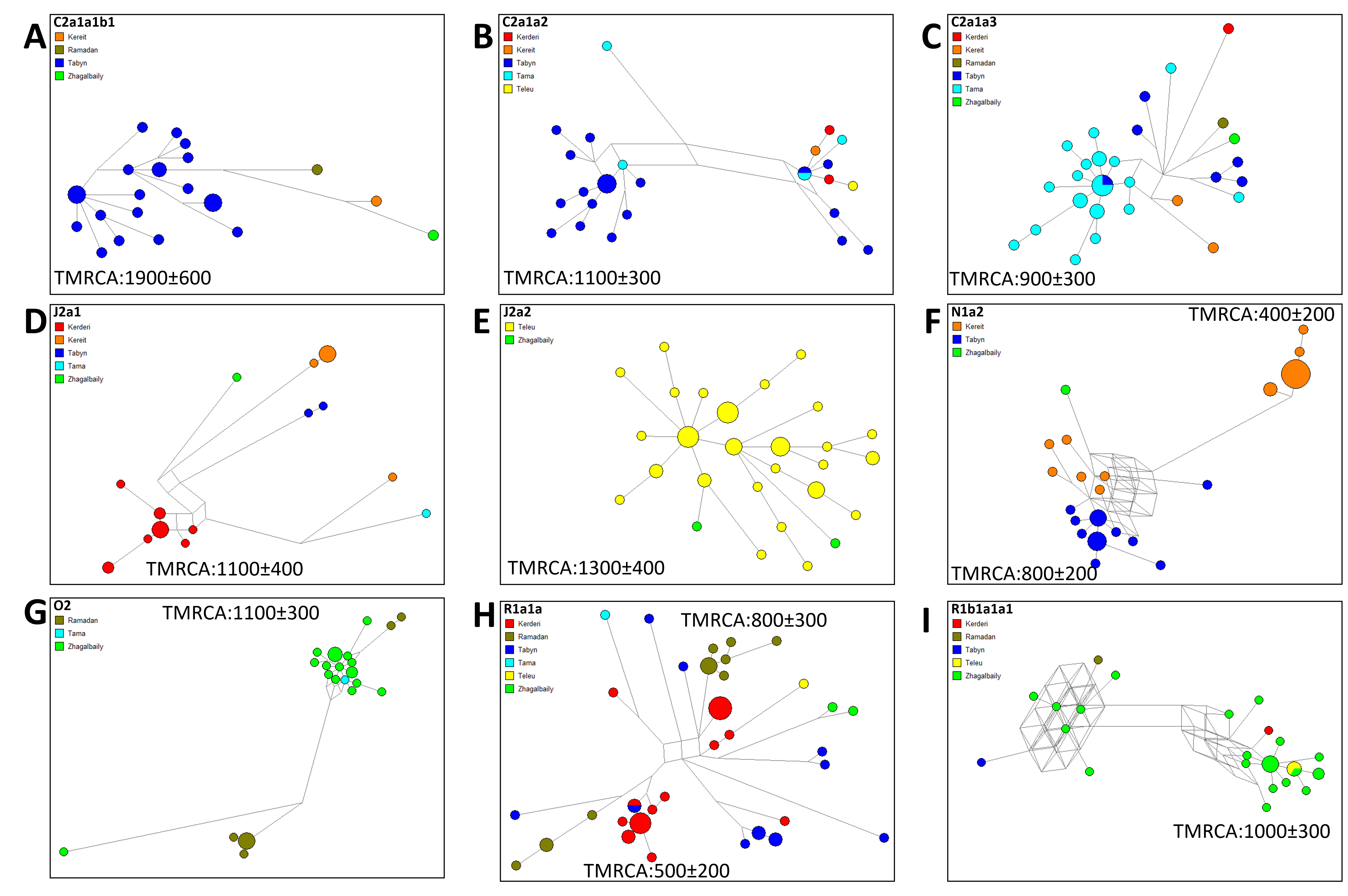

Supplement: Supplementary file 2 [file Image1.TIF]
